# Phenotypic and proteomic differences in biofilm formation of two *Lactiplantibacillus plantarum* strains in static and dynamic flow environments

**DOI:** 10.1016/j.bioflm.2024.100197

**Published:** 2024-04-22

**Authors:** Linda Huijboom, Parisa Rashtchi, Marcel Tempelaars, Sjef Boeren, Erik van der Linden, Mehdi Habibi, Tjakko Abee

**Affiliations:** a Food Microbiology, Wageningen University, Wageningen, 6708WG, the Netherlands; b Physics and Physical Chemistry of Foods, Wageningen University, Wageningen, 6708WG, the Netherlands; c Biochemistry, Wageningen University, Wageningen, 6708WG, the Netherlands

**Keywords:** Hydrophobicity, Nutrients, Enzyme treatments, Disinfectant resistance, Flow velocity

## Abstract

*Lactiplantibacillus plantarum* is a Gram-positive non-motile bacterium capable of producing biofilms that contribute to the colonization of surfaces in a range of different environments. In this study, we compared two strains, WCFS1 and CIP104448, in their ability to produce biofilms in static and dynamic (flow) environments using an in-house designed flow setup. This flow setup enables us to impose a non-uniform flow velocity profile across the well. Biofilm formation occurred at the bottom of the well for both strains, under static and flow conditions, where in the latter condition, CIP104448 also showed increased biofilm formation at the walls of the well in line with the higher hydrophobicity of the cells and the increased initial attachment efficacy compared to WCFS1. Fluorescence and scanning electron microscopy showed open 3D structured biofilms formed under flow conditions, containing live cells and ∼30 % damaged/dead cells for CIP104448, whereas the WCFS1 biofilm showed live cells closely packed together. Comparative proteome analysis revealed minimal changes between planktonic and static biofilm cells of the respective strains suggesting that biofilm formation within 24 h is merely a passive process. Notably, observed proteome changes in WCFS1 and CIP104448 flow biofilm cells indicated similar and unique responses including changes in metabolic activity, redox/electron transfer and cell division proteins for both strains, and myo-inositol production for WCFS1 and oxidative stress response and DNA damage repair for CIP104448 uniquely. Exposure to DNase and protease treatments as well as lethal concentrations of peracetic acid showed highest resistance of flow biofilms. For the latter, CIP104448 flow biofilm even maintained its high disinfectant resistance after dispersal from the bottom and from the walls of the well. Combining all results highlights that *L. plantarum* biofilm structure and matrix, and physiological state and stress resistance of cells is strain dependent and strongly affected under flow conditions. It is concluded that consideration of effects of flow on biofilm formation is essential to better understand biofilm formation in different settings, including food processing environments.

## 1 Introduction

*Lactiplantibacillus plantarum* (formally *Lactobacillus plantarum* [1]) is a Gram-positive, non-motile, rod-shaped species of lactic acid bacteria with various applications in the food industry, namely as a starter culture for fermented dairy-, meat- and plant-based products such as wine, cheeses, sauerkraut, and sausages and even as a probiotic [[2], [3], [4]]. Furthermore, substances produced by *L. plantarum* such as capsular or extracellular polysaccharides have the potential to be used as prebiotics [5]. Besides the beneficial uses of *L. plantarum*, the organism can also cause problems in the food industry as a spoilage organism for products such as beer [6], sliced meats [7], dressings and sauces [8], wine, cider and pickles [9]. Spoilage of food products leads to economic and qualitative loss for the food industry and consumers. According to a report in 2011 of the United Nations, one-third of the food generated for human consumption is either spoiled or wasted [10]. Furthermore, 17 % of the total global food production was still estimated to be wasted in 2021 [11]. As sustainability is becoming increasingly important, minimalizing the risk of food spoilage and subsequent food waste is of high importance.

One way that this spoilage organism can potentially contaminate food products is via the production of a biofilm [12]. Biofilms are layers of cells attached to a surface and surrounded by an extracellular matrix composed of polymeric substances including polysaccharides and extracellular DNA (eDNA). In the classical model of biofilm formation based on studies with motile, extracellular polysaccharide producing Gram-negative bacteria, e.g. *Pseudomonas* spp. [13,14], motile bacteria first adhere to a surface, followed by irreversible attachment. Next, they grow out into a microcolony and then start producing extracellular material that supports further growth and maturation of the biofilm. Under flow conditions and dependent on a range of biological and physical factors, a fraction of motile cells may actively detach from the biofilm, while other cells, or clumps of cells, are released due to shear stress. For biofilms in food processing environments that contain spoilage bacteria and/or pathogens, this may lead to food spoilage or food safety issues [12]. An additional risk may be caused by the previously observed increased resistance of biofilm cells to sanitization procedures mediated by protective and shielding effects of the matrix structure and/or components and/or intrinsic stress defence mechanisms [15]. These defence mechanisms can be activated under stress conditions encountered inside the biofilm including nutrient depletion and accumulation of toxic metabolites [16,17]. Enhanced survival of biofilm cells may thus enhance re-contamination and the outgrowth efficacy of spoilage and pathogenic bacteria [18]. It should be noted that, since *L. plantarum* also has beneficial qualities as a starter culture, the increased resistance of biofilm cells may also provide valuable opportunities that are relevant for fermentation processes, such as during wine production [19], and for the production of probiotics [20].

Biofilms vary greatly in both composition and structure, depending on the microorganism and environmental conditions [16,21]. *L. plantarum* biofilm studies are mostly done in static conditions [[22], [23], [24], [25]], as it is a non-motile organism. Static biofilm formation by *L. plantarum* has been studied in both single- and multi-strain biofilms [26]. Static biofilm formation by non-motile bacteria like *L. plantarum*, was previously described as a ‘passive’ process in which the initial attachment of the cells relies on sedimentation and/or changes in the cell surface to increase adhesion to surfaces [27]. For L. *plantarum*, various factors have already been identified to be important for biofilm formation such as the addition of manganese and glucose, which increases biofilm formation for model strain WCFS1 and other food-isolated strains [23]. In addition, comparative studies using *L. plantarum* wild type and capsular polysaccharide mutants showed that cell surface composition as well as cell lysis were found to be important factors involved in static biofilm formation [28]. For L. *plantarum* the extracellular biofilm material largely consists of eDNA as well as proteins and/or proteinaceous material [23]. The enhanced survival of *L. plantarum* static biofilm cells compared to planktonic cells was reported previously, including increased resistance to food related organic acids [29].

Although L. *plantarum* biofilm formation is mostly studied in static environments, the pipelines and equipment used in a food factory are highly dynamic. Fluid flow has a significant impact on biofilm formation by influencing various aspects such as microbial adherence, colonization, structure, nutrient supply, chemical signalling, and mechanical stress [30]. Research on biofilm formation under flowing conditions is limited for *Lactiplantibacillus*, but includes attachment efficacy and biofilm formation on polystyrene, silicone rubber and glass surface(s) [31,32]

We recently studied *L. plantarum* biofilm formation under static conditions and at four different flow rates using an in-house designed flow system [33]. The system consists of wells with inlets and outlets for injecting and removing the culture medium and creating a non-uniform velocity profile in the wells, resembling for example flow profiles in a corner or in a cavity on the surface of a pipe system. Our previous work primarily focussed on the influence of various flow speeds on biofilm formation of *L. plantarum* WCFS1 and CIP104448. Whilst in the current study, we used a comparative phenotyping and proteomics approach to identify factors contributing to biofilm formation under flow conditions using *L. plantarum* WCFS1 and CIP104448. This included quantification of static and flow biofilm cell numbers by plating (culturable cell numbers) and by quantification of live versus dead cells using flow cytometry in combination with cell-permeant SYOT9 and cell-impermeant Propidium-iodide (PI) nucleic acid stains, respectively. In addition, the biofilms were visualised using fluorescence and scanning electron microscopy (SEM), and their resistance to a disinfectant was quantified before and after dispersal. Linking phenotypes of the two strains to respective proteomes offered insight into similar and unique responses in biofilm formation under flow conditions.

## 2 Materials and methods

### 2.1 Bacterial strains and culture conditions

In this study, we used two strains of *Lactiplantibacillus plantarum* (*L. plantarum* WCFS1 and *L. plantarum* CIP104448). *L. plantarum* WCFS1 was isolated from human saliva [23], and *L. plantarum* CIP104448 was obtained from human stool. An overnight culture (OC) of each strain was prepared by inoculating cells from a −80 °C stock in 10 ml of Man-Rogosa-Sharpe (MRS) broth (Merck, Darmstadt, Germany) and incubated for 18 h at 30 °C. The OC was set to an optical density (OD) OD_600_ = 5 with fresh MRS and used to inoculate the culture medium consisting of 10-fold diluted brain heart infusion (BHI) (Becton, Dickinson, France) supplemented with 2 % glucose (Merck) and 0.005 % manganese sulphate (Merck), to an initial cellular concentration of approx. 7 log_10_ for both strains.

### 2.2 Formation of static and flow biofilms

Selected wells in a 48-well microtiter plate (Greiner bio-one, Alphen aan den Rijn, The Netherlands) were filled with 800 μl of culture medium. Each well was inoculated with 12 μl of OC (OD_600_ = 5). Next, the plate was placed in an incubator (New Brunswick™ Innova) at 30 °C and connected to an in-house designed system as described by Rashtchi et al.,] for continuous flow in selected wells [33]. The continuous flow had a flow rate of 3.2 ml/h, which corresponded to 4 volume changes per hour. This system can mimic a corner or a cavity in a pipe flow system, where part of the liquid can remain and part of the liquid can wash over. Different selected wells on the same 48-well plate were simultaneously used for static biofilm formation (flow rate of 0 ml/h). Plates were incubated for 24 h at 30 °C.

### 2.3 Quantification of biofilm using crystal violet

Crystal violet (CV) staining, as previously described by Fernández Ramirez et al.[23], was used to quantify the total amount of biofilm produced. Briefly, the supernatant of each well was removed gently using a pipette. Next, wells were rinsed three times with 900 μl of phosphate-buffered saline (PBS; pH 7.4 (Merck), NaCl 8 g/l, KCl 0.2 g/l, Na_2_HPO_4_ 1.44 g/l, and KH_2_PO_4_ 0.24 g/l). Remaining biofilm was stained for 30 min at room temperature with 800 μl of 0.1 (w/v) of CV (Merck). Excess CV was removed by rinsing the wells three times with 900 μl of PBS. The retained CV was then solubilized in 800 μl of 70 % ethanol for 45 min and repeated twice if needed, to solubilize all CV. Afterward, 200 μl of dissolved CV was transferred to a 96-well microplate and quantified by measuring the absorbance at 595 nm in a microplate reader (SpectraMax, Molecular Device, San Jose, USA). In the case of repeated solubilization of the CV, the values were measured independently and added together for the final OD value. Dilutions were made for values against the upper measuring limit of the Spectramax (>3.0) and blanks were subtracted. Data of at least three biological repetitions was used for analysis.

### 2.4 Enumeration of planktonic and biofilm cell numbers

Plate counting was used to determine the number of culturable cells in the static supernatant, static biofilm and flow biofilm grown in 48-well plates. Supernatant was collected by pipetting and wells were washed three times with 900 μl PBS. Attached cells (after 0, 15 and 30 min) and biofilm cells along with the biofilm matrix (24 h) were scraped off the well walls and bottom using a sterile pipet tip and resuspended in 1 ml PBS. Serial dilutions were done in peptone physiological salt solution (PPS) (Tritium-microbiology, Eindhoven, The Netherlands) and appropriate diluted samples were plated on MRS agar (Merck) followed by 48 h of incubation at 30 °C before counting. Data of three biological repetitions were used for analysis.

### 2.5 Protein quantification

Cells were gathered as described in “2.4" in 2 ml low binding Eppendorf tubes (Eppendorf, Hamburg, Germany) and pelleted by centrifuging (Eppendorf Centrifuge 5804 R) at 17,000 g for 5 min at 4 °C. To prepare the samples for analysis, the cell pellet was rinsed twice with 200 μl of cold 100 mM Tris buffer (pH 8; Sigma-Aldrich, Burlington, USA). The samples were then frozen and kept at −80 °C until needed. Prior to analysis, the cell pellets were defrosted, resuspended in 50 μl of 100 mM Tris buffer with a pH of 8, and subjected to three rounds of sonication using a Soniprep 150 sonication probe (MSE, UK) on ice, each lasting 15 s, with vortexing in between. Lysis of all cells was confirmed by plating of a test sample and subsequently no outgrowth was observed. The protein concentration was determined using the Bicinchoninic Acid (BCA) method [34]. Biological duplicates were used for analysis.

### 2.6 Bacterial hydrophobicity

Bacterial hydrophobicity was evaluated by measuring microbial adhesion to *n*-hexadecane (MATH) as a solvent, following [35]), with slight modifications. An OC of both strains was prepared by inoculating cells from a −80 °C stock in 10 ml of 10-fold diluted BHI supplemented with 2 % glucose and 0.005 % manganese sulphate and incubated for 18 h at 30 °C.The OCs of both strains were centrifuged at 8000 g for 5 min to pellet the cells. Next, the cell pellets were resuspended in PBS to a final OD_600_ of 0.3–0.4 and 4 ml of this suspension was combined with 1 ml of *n*-hexadecane. Samples were vortexed for 1 min and then incubated at room temperature for 30 min until a phase separation was visible. The optical density of the aqueous phase was measured at 600 nm. The percentage of affinity to *n*-hexadecane (i.e. hydrophobicity) was expressed using the following equation:

[OD_600_ (original bacterial suspension)-OD_600_ (aqueous phase)/OD_600_ (original bacterial suspension)]*100.

In this procedure, the more hydrophobic cells will move to the *n*-hexadecane phase which causes lowering of OD in the water phase. Data of three biological replicas with each at least two technical replicas was used for analysis.

### 2.7 Quantification of live, dead and damaged cells using flow cytometry

The portion of live, dead and damaged cells in the static supernatant and the formed biofilms under static and flow conditions, was measured using flow cytometry. Samples were gathered as described in “2.4”. Supernatant and resuspended biofilm samples were centrifuged (16,000 g, 5 min, 4 °C) to create a cell pellet. Extracellular DNA was removed by adding 200 μl of 100 μg/ml DNase I (final concentration in PBS) (Sigma-Aldrich, Burlington, USA) and incubated at 30 °C for 1 h, followed by a two-time wash-up with PBS to remove the remaining enzyme. Next, cells were resuspended in 1 ml PBS and live/dead cell staining was done based on membrane integrity and nucleic acid stains. In this work, SYTO9 and PI were utilized as DNA binding fluorophores. Green fluorescent SYTO9 can enter all cells, while red fluorescent PI only enters cells with compromised cytoplasmic membranes. Therefore, SYTO9 (green) stained cells were interpreted as live cells, PI (red) stained cells as dead cells, and double stained cells as damaged. The cells were stained with 1 μl SYTO9 (0.0334 mM) and 1 μl PI (0.2 mM). A FACSAria III (BD Biosciences, Franklin Lakes, USA) was used to analyse 50,000 events per sample. A 488-nm laser was used to measure the forward scatter (FSC)/side scatter (SSC) parameters and SYTO9 (502LP and 530/30 filter set). PI was measured using a 561-nm laser with a 600LP and 610/20 filter set. First, single cells were gated based on FSC/SSC and positive staining with SYTO9. Second, the correct setup for PI was established using a heat inactivated (15 min at 70 °C) cell sample as a positive stain control and a healthy culture as a negative PI stain control. Finally, the percentage of PI stained cells was determined using FlowJO 10 (FlowJo™, Ashland, USA) to analyse the flowcytometry data. Three biological replicas were used for further analysis.

### 2.8 Fluorescence microscopy and enzymatic treatments

Samples were gathered as described in “2.4”. Supernatant and resuspended biofilm samples were centrifuged for 5 min at 8000 g. Cell pellets were resuspended in 500 μl PBS and stained with 0.0334 mM SYTO9 and 0.2 mM PI. The excitation/emission maxima of these dyes are ∼480/500 nm for SYTO9 and ∼490/635 nm for PI. After staining, 5 μl of the cell suspension was placed on a 2 % agarose pad on a glass slide. Fluorescence microscopic images were made using an Axioskope epifluorescence microscope (Carl Zeiss, Germany) equipped with at 50-W mercury lamp, a carboyfluorescein (cF) and diacetate (cFDA) filter set (excitation wavelength 450–490 nm, emission wavelength >500 nm), an ×100 Plan-Neofluar objective lens, and a camera (Carl Zeiss, Germany). For the samples that were treated with enzymes, prior to adding the dyes, the samples were treated by resuspending the obtained pellet in either 1 ml of 100 μg/ml DNase I solution (final concentration in PBS), or a solution with 10 μg/ml Proteinase K (Qiagen, Hilden, Germany), or 1 ml PBS (as a control) and incubated at 30 °C for 1 h. After the treatment, cells were pelleted and washed three times with PBS before adding the dyes, followed by fluorescence microscopy analysis as described.

### 2.9 Scanning electron microscopy

For the Scanning Electron Microscopy (SEM) images, the biofilms were grown as described above in "2.2", except a clear sterile ∼0.8 × 0.8 cm polystyrene coupon was attached to the bottom of each well with Vaseline. After washing of the biofilms the coupons were removed from the wells with tweezers. Coupons were prepared for SEM imaging by fixating for 1 h with 2.5 % glutaraldehyde in 0.1 M phosphate/citrate buffer, followed by 6 washing steps of 10 min with 0.1 M phosphate/citrate buffer. Next 1 % osmium tetroxide in 0.1 M phosphate/citrate buffer was added and incubated for 1 h followed by three washing steps of 10 min in MilliQ water. After fixation, the samples were dehydrated by submerging them in a series of ethanol solutions (30, 50, 70, 80, 90, and 96 % (v/v)) for 5 min, followed by submersion in 100 % ethanol twice for 10 min. The samples were further dried using a critical point drier with 100 % ethanol to preserve the surface structure. The dried samples were attached to aluminium stubs with double sided carbon stickers and sputter coated with 12 nm tungsten. Finally, the samples were visualised using a scanning electron microscope (Magellan 400, FEI) at magnifications of 2500*X* up to 25000*X* with secondary electron detection of 2.00 kV and 13 pA to analyse the surface of the coupons.

### 2.10 Disinfection treatments

Cells were grown as described in “2.2”. Planktonic cells, cells within the biofilm, and cells originated from resuspended biofilm were exposed to 20 μg/ml peracetic acid (PAA) for 12 min at room temperature under static conditions. Planktonic cells from the supernatant were collected by centrifugation of the supernatant for 5 min at 8000 g and washed three times with PBS. The final pellet was resuspended in 1 ml of disinfectant agent for 12 min. For the treatment of both static and flow biofilms, the biofilms were washed twice with PBS and exposed to disinfectant agent for 12 min. Next, the biofilms were washed once with PBS and resuspended in 1 ml PBS. For cells originated from resuspended biofilm, the washed biofilms were scraped from the well surface using a sterile pipette tip and resuspended in 1 ml of disinfectant agent, and incubated at room temperature for 12 min. After treatments, the cells were serially diluted in PPS and plated on MRS agar plates, followed by incubation at 30 °C for 48 h. Data of three biological replicas with each at least two technical replicas were used for analysis.

### 2.11 Proteome sample preparation and analysis

Supernatant and biofilm cells were gathered for proteomic analysis using the protein aggregation capture (PAC) method [36]. Cells were gathered and protein concentration was determined as described in “2.5”, and the protein concentration was set to ∼50 μg per sample. Preparation of the samples was done as described by Huijboom et al.[37,]). Samples were examined in biological triplicates and each protein sample was analysed by injecting 5 μl into a nano-Liquid Chromatography (LC)1000-Exploris 480 Mass Spectrometry (MS)/MS as described before [38]. LCMS data was analysed using the MaxQuant quantitative proteomics software package [39] and a *L. plantarum* WCFS1 (NCBI 220668) and CIP14448 (NCBI ASM95619v1) database using settings as described [40]. Data of the mass spectrometry proteomics has been deposited to the ProteomeXchange Consortium via the PRIDE [41] partner repository with the dataset identifier PXD047751.

### 2.12 Proteome data analysis

The normal logarithm was taken from protein LFQ MS1 intensities as obtained from MaxQuant. Zero “Log LFQ” values were replaced in Perseus [42] by a value taken of a normal distribution using default down shift (1.8) and width (0.3) values to make sensible ratio calculations possible. In Perseus samples were grouped and two sample T tests were performed using the “LFQ intensity” columns obtained with (permutation based) FDR set to 0.05 and S0 set to 1. Next, Rstudio was used to create Venn-diagrams, cut-off lists and volcano plots using the following packages: ‘dplyr’ [43], ‘tidyverse’ [44], ‘ggplot2’ [45], ‘ggrepel’ [46] and ‘ggVennDiagram’ [47]. An ortholog comparison was created using the protein sequences available in NCBI. Protein changes were deemed significant if p < 0.05 and >1.5-fold change.

### 2.13 General data analysis

Data of all experiments was gathered in Excel and further analysed and visualised in Rstudio unless stated otherwise. Significance was determined using two sample T tests in Rstudio and samples were considered significant if p < 0.05.

## 3 Results

### 3.1 Biofilm formation and quantification of cell numbers by plate counting

**Fig. 1:**
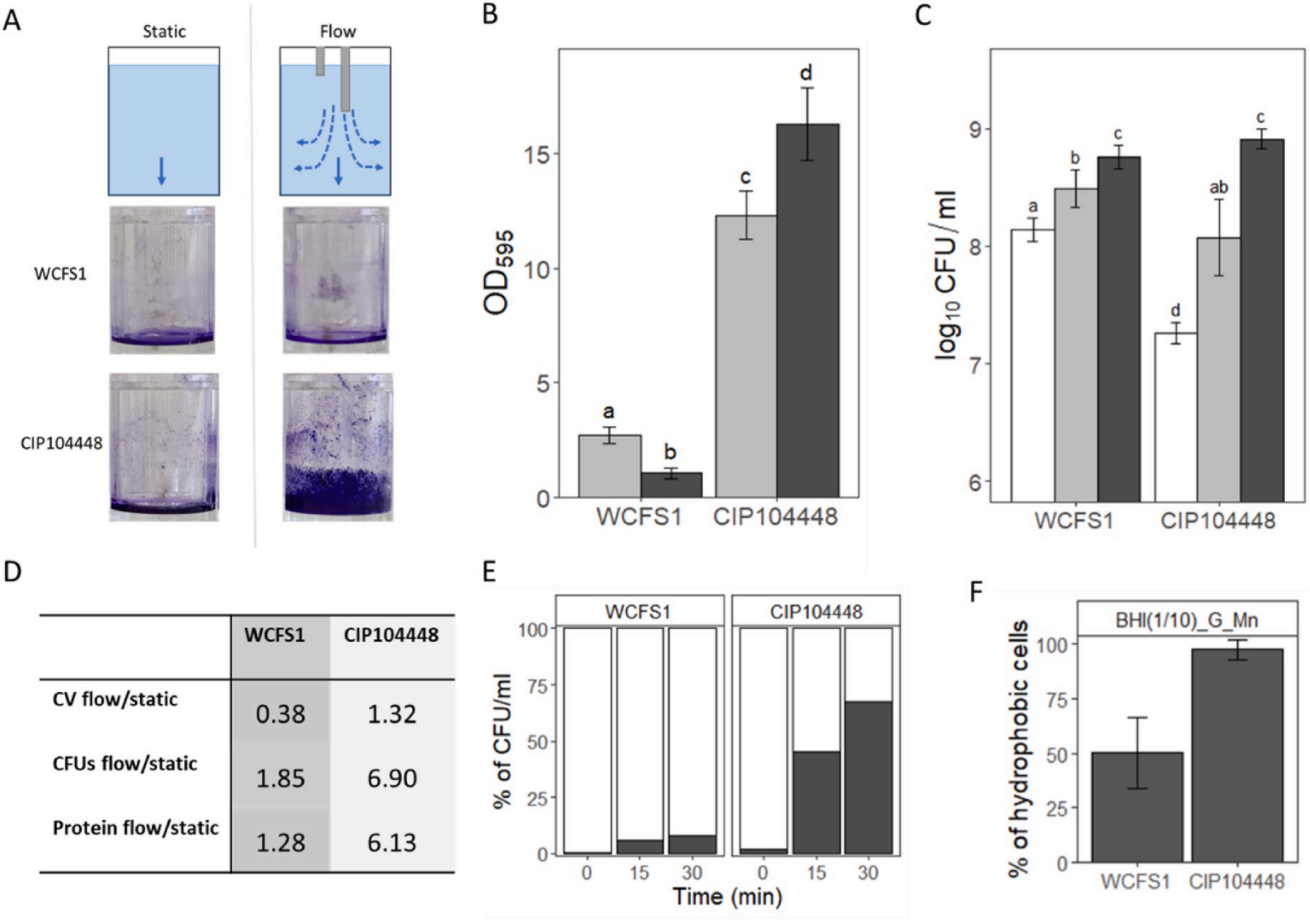
A) Schematic illustration of the static and flow biofilm formation model in each well (top row) with corresponding CV biofilm staining of *L. plantarum* WCFS1 (middle) and CIP104448 (bottom) in static condition (left side) and flow (right side). B) Dissolved crystal violet (CV) as indication for biofilm formation for WCFS1 and CIP104448. Light grey indicates biofilm formed in a 48-well plate (24 h, 30 °C) during static incubation and dark grey during incubation with a flow of 3.2 ml/h. Different letters indicate statistically significant differences. C) Colony forming units found in static supernatant (white), static biofilm (light grey) and biofilm grown under flow (dark grey) for WCFS1 and CIP104448. Different letters indicate statistically significant differences. D) Ratios comparing flow and static biofilm cells based on CV, CFU and protein content. E) Attachment of cells to the polystyrene well plate over time in percentages. White indicates the supernatant and dark grey the attached/biofilm cells. The cell counts of both the supernatant cells and attached cells at each time point constitutes to the total CFU/ml (100 %). F) Hydrophobicity of cells in 1/10 BHI with 2 % glucose and 0.005 % manganese sulphate. (For interpretation of the references to colour in this figure legend, the reader is referred to the Web version of this article.)

Crystal violet staining for both visualisation and quantification of the biofilm, along with determination of cell numbers in both biofilms and static supernatant generated by *L. plantarum* WCFS1 and *L. plantarum* CIP104448 under flow and static conditions was determined (Fig. 1A–C). For WCFS1, the quantified CV of biofilm formed under flow is reduced compared to that in static conditions, while increased biofilm formation under flow was observed for CIP104448 (Fig. 1B). The CV images in Fig. 1A showed that the biofilm was formed at the bottom of the wells for WCFS1 under both static and flow conditions. However, for CIP104448, the static biofilm was located at the bottom of the well, but the flow-induced biofilm formation was also observed on the walls of the well. The cell numbers in the static supernatant were lower for both strains compared to the biofilms (Fig. 1C). Furthermore, the cell numbers in WCFS1 supernatant are approximately 1.1 log_10_ CFUs/well higher compared to that of CIP104448. As the biofilms were resuspended in 1 ml, the concentrations in CFUs/ml indicated in Fig. 1C correspond to the overall number of cells in the entire biofilm, which clearly increased under flow conditions for both strains. In WCFS1 and CIP104448, the increase between static and flow conditions is 0.3 and 0.8 log_10_ CFU/well, respectively.

### 3.2 Biofilm CV, CFU and protein ratios

Fig. 1D shows the flow-to-static condition ratio for CV value, CFU value, and protein production for each strain. The flow-to-static ratio for CV staining was 0.38 for WCFS1 and 1.32 for CIP104448, while the flow-to-static ratio for cell counts is 1.85 and 6.90 fold in WCFS1 and CIP104448, respectively. The protein in the biofilm matrix and the protein inside the bacteria make up the total amount of protein in the biofilm. Our findings show that for both WCFS1 and CIP104448, the protein ratio between flow and static biofilms is in line with the CFU ratio, namely 1.28 and 6.13 for WCFS1 and CIP10448, respectively.

### 3.3 Cell hydrophobicity and surface adhesion

The number of cells that adhered to the polystyrene surface of both strains was not comparable. After 30 min, significantly more CIP104448 cells were attached to the surface compared to WCFS1 (Fig. 1E), which may be attributed to the higher hydrophobicity of CIP104448 cells (Fig. 1F). The values in percentages indicate that CIP104448 cells were more hydrophobic than WCFS1 cells, with 97 % of cells in the hydrophobic fraction, compared to 50 % for WCFS1. The hydrophobicity of both strains was also determined in the OC media (MRS) and was in line with the results obtained in 1/10 BHI with 2 % glucose and 0.005 % manganese sulphate (Supplementary material S1).

### 3.4 Flow cytometry analysis

**Fig. 2:**
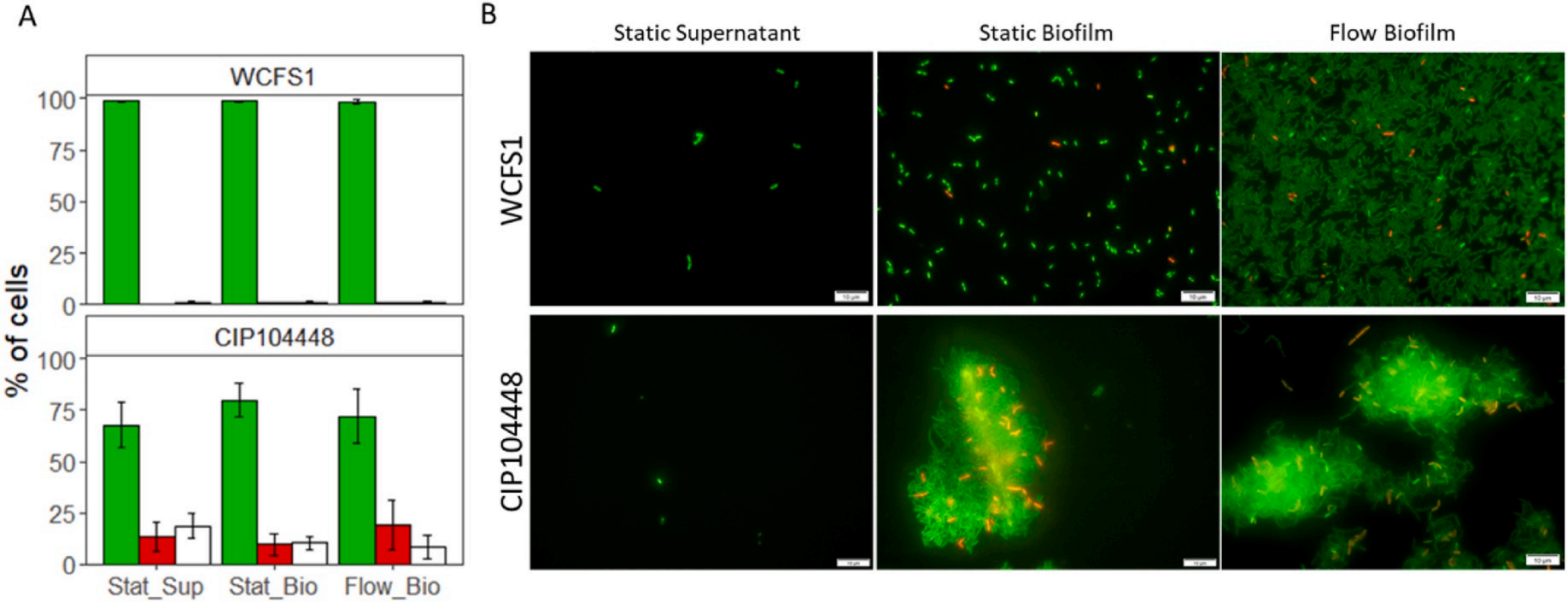
A) Percentages of *L. plantarum* WCFS1 and CIP104448 cell cultures stained with SYTO9 (green), PI (red) or both (white) measured using flow cytometry. Cell cultures used where static supernatant cells (Stat_Sup), static biofilm cells (Stat_Bio) and biofilm cells grown under 3.2 ml/h flow (Flow_Bio). B) Corresponding fluorescence images stained with SYTO9 and PI resulting in green and red fluorescence respectively. Scale bars are indicated in the images and represent 10 μm. (For interpretation of the references to colour in this figure legend, the reader is referred to the Web version of this article.)

Flow cytometry was utilized to determine the proportion of live, dead, and damaged cells in the biofilms of two strains under flow and static conditions, as well as in the static supernatant. The preponderance of live cells in WCFS1 samples is clearly shown in Fig. 2A, with >98 % of live cells and fewer than 2 % dead or damaged cells. In contrast, around 30 % of cells in the CIP104448 strain were dead or damaged in all three conditions tested. Furthermore, the proportion of live cells in the biofilms and the supernatant was not significantly different. These results are consistent with the fluorescence images (Fig. 2B), where the proportion of dead cells to live cells in the CIP104448 strain is higher than in WCFS1. Furthermore, WCFS1 fluorescence images demonstrated a greater number of SYTO9-stained live cells in the flow biofilm compared to static conditions, which is in line with the observed cell numbers determined by plate counts (Fig. 1C). The static supernatant, static biofilm and flow biofilm of both strains were also exposed to DNAse I and Proteinase K, which showed that the neither enzymatic treatment dissolved the cell aggregates formed by CIP104448 (Supplementary material S2).

#### 3.4.1 Scanning electron microscopy (SEM)

**Fig. 3:**
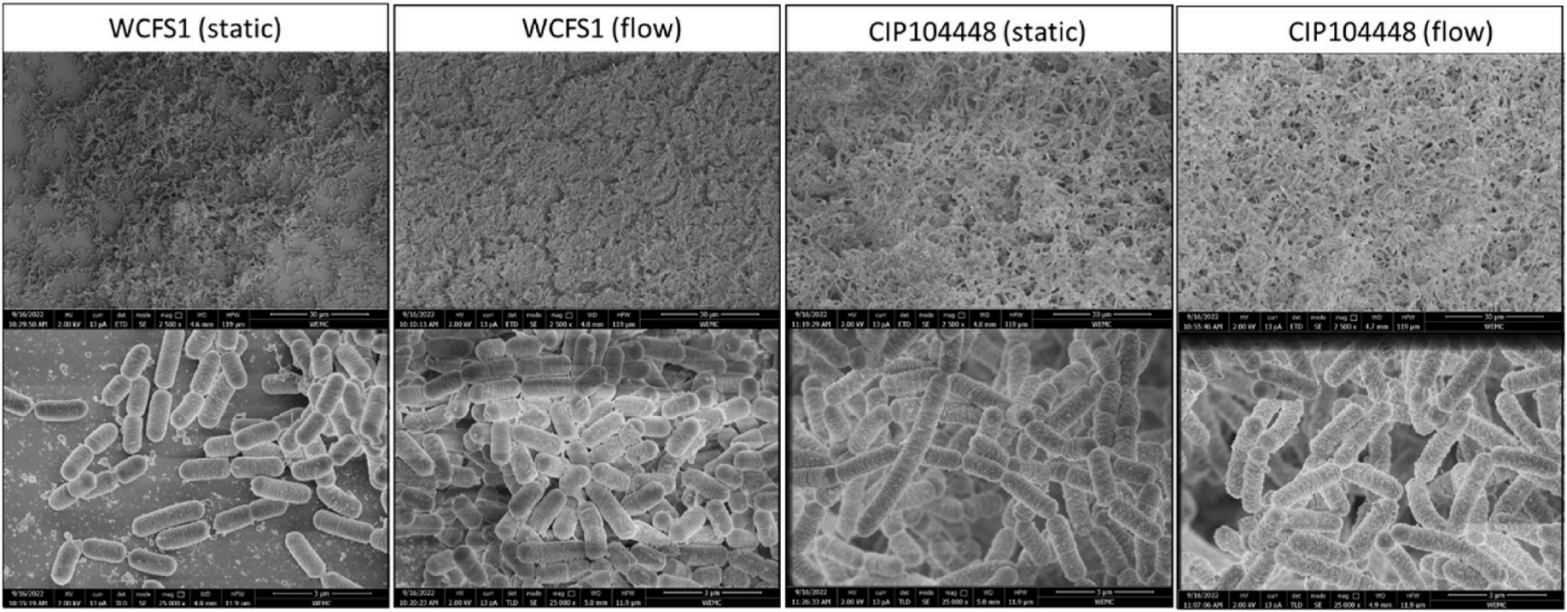
Scanning electron microscopy images of *L. plantarum* WCFS1 and CIP104448 biofilm cells grown on polystyrene coupons either static or with 3.2 ml/h flow at two magnifications. Scale bars are indicated in the images and represent 30 μm in the upper row and 3 μm in the lower row.

SEM was used to better visualize the developed biofilms. In both conditions for the CIP104448 strain, the SEM images (Fig. 3) showed that the structure of the biofilm was formed by clusters of agglomerated cells in a 3D structure, which is consistent with Fig. 2 (B). For WCFS1, the cells adhered closer to each other and formed a closed packed layer. Additionally, CIP104448 biofilm cells appear longer than WCFS1 biofilm cells.

### 3.5 Disinfectant treatments

**Fig. 4:**
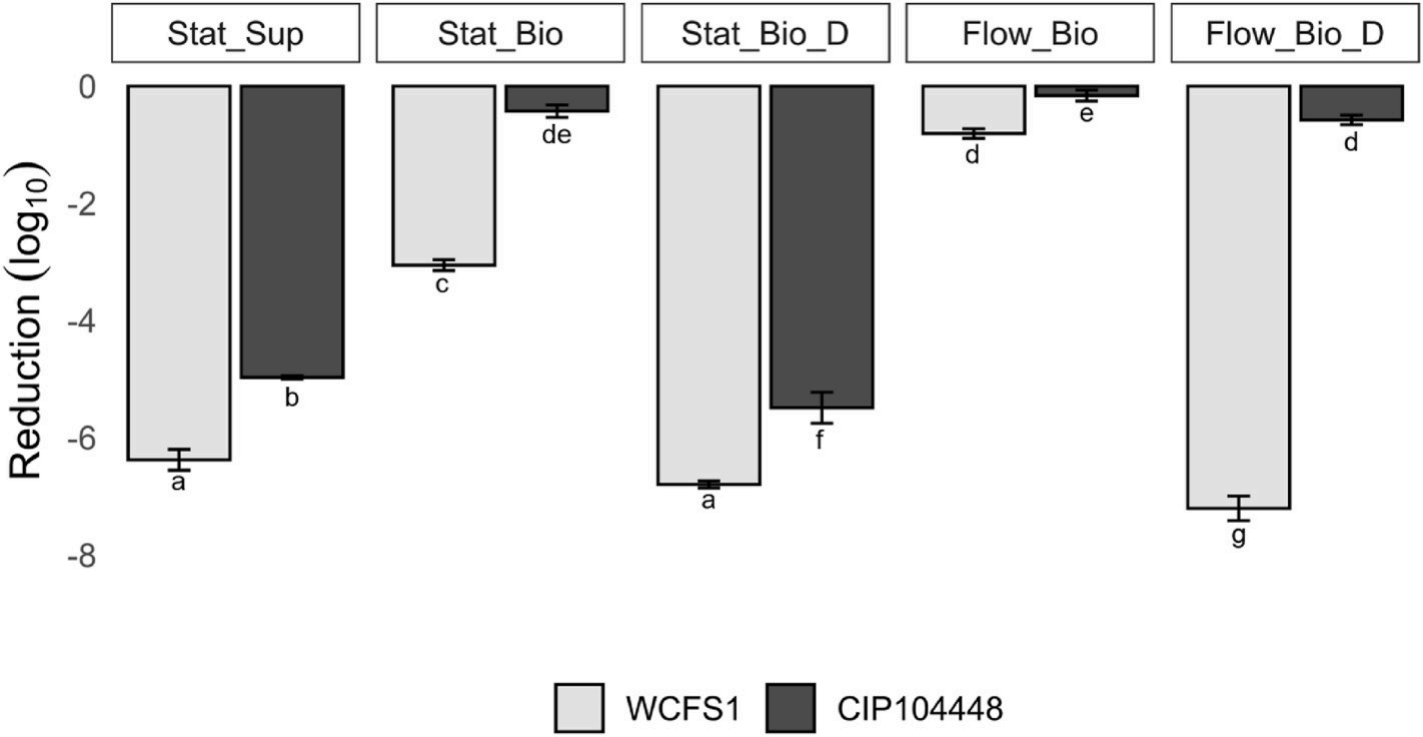
Effect of peracetic acid (20 μg/ml) on static supernatant (Stat_Sup), static biofilm (Stat_Bio), static biofilm dispersed (Stat_Bio_D), flow biofilm (Flow_Bio) and flow biofilm dispersed (Flow_Bio_D) cells, respectively. Light grey is the cell count reduction measured after treatment for WCFS1 and dark grey for CIP104448. Different letters indicate statistically significant differences.

Planktonic cells from the static supernatant, cells within the biofilms, and cells derived from resuspended biofilms were exposed to the disinfectant PAA. Fig. 4 depicts the decrease in cell numbers after disinfectant treatment. Our results showed that cells in the intact static and flow biofilms of both strains were more resistant to the disinfection treatment than planktonic cells from the supernatant. Between the two biofilms, cells of the intact flow biofilm were more resistant compared to cell of the intact static biofilm, for WCFS1, but no significant difference was found for CIP104448. Dispersed cells of the static biofilms of both strains showed similar sensitivity to the treatment compared to planktonic cells. However, the sensitivity of dispersed biofilm cells grown under flow was strain specific and showed increased sensitivity for WCFS1 compared to undispersed cells, but minimal influence on CIP104448. Overall, CIP104448 biofilms were more resistant to the disinfectant treatment compared to WCFS1.

### 3.6 Comparative proteomics

**Fig. 5:**
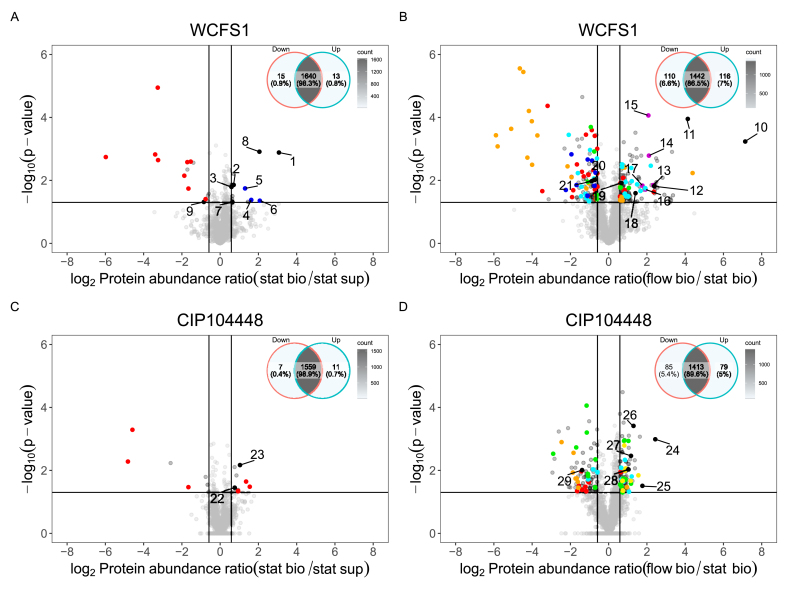
Volcano plots of WCFS (A and B) and CIP104448 (C and D) proteomes of static biofilm vs static supernatant (A and C) and flow biofilm vs static biofilm (B and D). Positive log_2_ fold change values indicate higher abundance in the first condition compared to the second. Negative values indicate a lower abundance. Each grey circle represents a unique protein. A cut off of 1.5-fold change and a p-value of 0.05 was used and is indicated by the black lines. Fully coloured circles indicate proteins related to various clusters with the following colour codes: purine/pyrimidine metabolism (orange), cell surface/adhesion (red), myo-inositol metabolism (Purple), redox/oxidoreductases (turquoise), stress (green), nutrients (dark blue), ribosomal proteins (yellow). Noteworthy proteins that did not fall within a colour group are indicated by black circles and numbers correspond to Table 1. (For interpretation of the references to colour in this figure legend, the reader is referred to the Web version of this article.)

For both strains, the static biofilm cells compared to static supernatant cells showed minimal differences in protein levels. For WCFS1, 13 proteins (0.8 %) were higher expressed and 15 proteins (0.9 %) showed lower expression levels in static biofilm cells compared to static supernatant cells (Fig. 5A). A full table of the protein changes can be found in Supplementary material S3, and a table with selected proteins corresponding to the volcano plots of Fig. 5 are shown in Table 1. The higher expressed proteins in the static biofilm were related to various (general) processes such as cysteine/methionine metabolism (F9URY4), sugar metabolism (F9ULK7, F9URE1 and F9UT00) and proteins related to magnesium (binding) (F9URJ9 and F9USN4), along with two transcription regulators of the TntR family (F9ULB4 and F9URB9). Of the lower expressed proteins in the static biofilm, nine out of 15 were related to the cell wall/surface (F9ULM2, F9UNC2, F9URS2, F9URU9, F9USE1, F9UTQ7, F9UU93, F9UUA0), including a protein related to adhesion (F9UP60, Table 2). Additionally, foldase protein PrsA2 (Q88T16) was also lower expressed. For CIP104448, 11 proteins (0.7 %) were higher expressed and seven proteins (0.4 %) were lower expressed in static biofilm cells compared to static supernatant cells (Fig. 5C). Similarly to WCFS1, no clear trend was observed in the higher expressed proteins, but two thioredoxin related proteins (WP_003641528.1 and WP_003642657.1) were found to be increased in the static biofilm cells (Table 1). Interestingly, of the 11 proteins that were differentially expressed, six were related to the cell wall. (Table 2). When comparing the cell wall related proteins of both strains in static biofilm cells versus static supernatant cells, four proteins overlapped in both strains, of which three had the same expression trend. Namely, the cell surface protein F9ULM2 and two of the extracellular transglucosylases with a LysM peptidoglycan binding domain (F9USE1 and F9UTQ7 were extremely lower expressed in both strains (−9.5 fold, −9.6 fold and −63.0 fold for WCFS, and −28.3 fold, −3.2 fold and −24.0 fold for CIP104448, respectively). In addition, one membrane protein (F9UNC2) was differentially expressed and expression went down in WCFS1 (−2.9 fold), but up in CIP104448 (1.7 fold). Overall, for both strains there were limited protein changes in the static biofilm compared to the static supernatant and the majority of the total retrieved proteins remained similar (98.3 % and 98.9 % for WCFS1 and CIP104448, respectively).

**Table 1:** Selected differential expressed proteins in static and flow biofilm cells of *L. plantarum* WCFS1 and CIP104448. Numbers correspond to numbers indicated in the volcano plots in Fig. 5.

WCFS1 static biofilm over static supernatant cells					
*# in* 5A	*Protein ID*	*Gene code*	*Annotation*	*Gene*	*Fold change*
1	F9URY4	*lp*_2888	Cystathionine beta-lyase	*cblA2*	**8.4**
2	F9ULB4	*lp*_3558	Transcription regulator, GntR family	*araR*	**1.6**
3	F9URB9	*lp*_2615	Transcription regulator, GntR family	*-*	**1.5**
4	F9ULK7	*lp*_3660	Ribokinase	*rsbK1*	**3.1**
5	F9URE1	*lp*_2648	PTS system, *N*-acetylglucosamine-specific EIID component	*pts19D*	**2.5**
6	F9UT00	*lp*_0189	Raffinose-6-phosphate hydrolase & stachyose-6-phosphate hydrolase	*-*	**4.2**
7	F9URJ9	*lp*_2723	Amidophosphoribosyltransferase	*purF*	**1.6**
8	F9USN4	*lp*_3123	NAD (+) diphosphatase	*-*	**4.2**
9	Q88T16	*lp*_3193	Foldase protein	*prsA2*	**−1.8**
**WCFS1 flow biofilm over static biofilm cells**					
# in 5B	*Protein ID*		*Annotation*		*Fold change*
10	F9USK0	*lp*_3085	Asparagine synthase (Glutamine-hydrolysing)	*asnB2*	**140.9**
11	F9URT8	*lp*_2830	Aspartate ammonia-lyase	*ansB*	**17.6**
12	F9UT54	*lp*_0256	Cystathionine beta-synthase	*cbs*	**5.2**
13	F9ULG2	*lp*_3608	Myo-inositol 2-dehydrogenase-like (Promiscuous)	*-*	**5.1**
14	F9ULF8	*lp*_3604	Myo-inositol (And similar sugars) transporter, major facilitator superfamily	*iolT1*	**4.3**
15	Q88S38	*lp*_3606	Inositol 2-dehydrogenase/D-chiro-inositol 3-dehydrogenase	*iolG*	**4.2**
16	F9ULG4	*lp*_3612	Myo-inositol 2-dehydrogenase-like (Promiscuous)	*-*	**3.8**
17	Q88S37	*lp*_3607	Inosose dehydratase	*iolE*	**3.4**
18	F9UP81	*lp*_1723	Hydrolase, HAD superfamily, Cof family	*-*	**2.6**
19	Q88X16	*lp*_1438	6,7-dimethyl-8-ribityllumazine synthase	*ribH*	**1.6**
20	Q88X05	*lp*_1452	Foldase protein	*prsA1*	**−1.7**
21	F9UU03	*lp*_0416	Two-component system histidine protein kinase PlnB sensor protein	*plnB*	**−1.9**
**CIP104448 static biofilm over static supernatant cells**					
*# in* 5C	*Protein ID*		*Annotation*		*Fold change*
22	WP_003641528.1	*-*	Thioredoxin	*trxA*	***1.7***
23	WP_003642657.1	*-*	Thioredoxin family protein	*trxH*	***2.1***
**CIP104448 flow biofilm over static biofilm cells**					
*# in* 5D	*Protein ID*		*Annotation*		*Fold change*
24	WP_045351456.1	–	Cysteine synthase family protein	–	**5.4**
25	WP_045352415.1	–	SWIM zinc finger family protein	–	**3.4**
26	WP_045351833.1	–	Riboflavin synthase	–	**2.5**
27	WP_045351835.1	–	Bifunctional 3,4-dihydroxy-2-butanone-4-phosphate synthase/GTP cyclohydrolase II	–	**2.2**
28	WP_045351832.1	–	Bifunctional diaminohydroxyphosphoribosylaminopyrimidine deaminase/5-amino-6-(5-phosphoribosylamino)uracil reductase	*ribD*	**2.1**
29	WP_003641528.1	–	Thioredoxin	*trxA*	**−2.6**

**Table 2:** Overview of differential expressed cell wall related proteins. Significant values are in bold. WCFS1 annotation was used. (*CIP104448 prot ID/annotation).

Static biofilm over static supernatant cells					
*Protein ID*	*Gene code WCFS1*	*Gene*	*Annotation*	*Fold change WCFS1*	Fold change *CIP104448*
F9UP60	*lp*_1697	*-*	Adherence protein, chitin-binding domain	**−3.2**	−1.9
F9URS2	*lp*_2809	*-*	Extracellular protein	**−3.3**	−1.2
F9URU9	*lp*_2847	*-*	Extracellular transglycosylase, with LysM peptidoglycan binding domain	**−10.5**	−1.9
F9UU93	*lp*_3414	*-*	Cell surface protein, CscB family	**−3.7**	NA
F9UUA0	*lp*_3421	*-*	Extracellular protein,gamma-d-glutamate-*meso*-diaminopimelate muropeptidase	**−2.9**	1.1
F9UTQ7	*lp*_0302	*-*	Extracellular transglycosylase	**−63.0**	**−24.0**
F9UNC2	*lp*_1357	*-*	Extracellular protein, membrane-anchored	**−1.7**	**2.9**
F9USE1	*lp*_3014	*-*	Extracellular transglycosylase, with LysM peptidoglycan binding domain	**−9.6**	**−3.2**
F9ULM2	*lp*_3679	*-*	Cell surface protein, CscB family	**−9.5**	**−28.3**
F9USJ3	*lp*_3077	*-*	Extracellular protein	−1.1	**2.6**
F9ULL9	*lp*_3676	*-*	Cell surface protein, CscC family	−1.2	**1.9**
***flow biofilm over static biofilm cells***					
Q88UU5	*lp*_2361	*murA1*	UDP-*N*-acetylglucosamine 1-carboxyvinyltransferase 1	**5.1**	1.4
F9UPJ6	*lp*_1866	*-*	Extracellular protein, DUF2140 family	**2.1**	**1.4**
F9UQ81	*lp*_2137	*ftsW*	Cell division protein	**1.8**	NA
F9UU44	*lp*_0461	*-*	Cell surface hydrolase, membrane-bound	**1.7**	**1.5**
F9UQF8	*lp*_2232	*-*	Hypothetical membrane protein, DedA family	**1.5**	1.4
F9UQQ6	*lp*_2354	*rodA2*	Rod-shape determining protein	**1.5**	1.0
F9UL10	*lp*_0536	*-*	Cell surface protein, ErfK/YbiS/YcfS/YnhG family	**−1.5**	−2.1
F9UNV5	*lp*_1577	*-*	Hypothetical extracellular protein	**−1.5**	−1.8
F9UU90	*lp*_3411	*-*	Extracellular protein, DUF1002 family	**−1.5**	1.0
F9URG0	*lp*_2671	*-*	Transport protein with CBS domains, DUF21 family	**−1.6**	**−1.9**
F9UP53	lp_1690	*-*	Hypothetical membrane protein, SPFH domain/Band 7 family	**−1.6**	**−1.6**
Q88W95	*lp*_1754	*gpsB*	Cell cycle protein	**−1.7**	1.3
F9UQM5	*lp*_2318	*mreC*	Cell shape-determining protein	**−1.7**	−1.7
F9UPL2	*lp*_1884	*-*	Extracellular protein, with LysM peptidoglycan binding domain	**−1.8**	−1.3
F9URZ3	*lp*_2901	*-*	Hypothetical membrane protein	**−2.1**	**−3.0**
F9UN23	*lp*_1229	*msa*	Mannose-specific adhesin, LPXTG-motif cell wall anchor	**−2.1**	NA
F9UQ85	*lp*_2145	*-*	Extracellular protein, cell-wall anchored	**−2.6**	**−2.7**
F9URD9	*lp*_2645	*Acm2*	Cell wall hydrolase/muramidase	**−3.2**	−2.2
F9UP14	*lp*_1643	*-*	Mucus-binding protein, LPXTG-motif cell wall anchor	**−3.6**	NA
F9ULM1	*lp*_3678	*-*	Cell surface protein, CscA/DUF916 family	**−3.8**	−1.8
F9US12	*lp*_2925	*-*	Cell surface protein, LPXTG-motif cell wall anchor	**−3.8**	**−4.2**
F9UP60	*lp*_1697	*-*	Adherence protein, chitin-binding domain	**−9.1**	NA
F9USJ9	*lp*_3084	*-*	Cell surface protein, ErfK family	**−11.1**	NA
F9USE1	*lp*_3014	*-*	Extracellular transglycosylase, with LysM peptidoglycan binding domain	**1.7**	**−2.7**
F9UUA0	*lp*_3421	*-*	Extracellular protein,gamma-d-glutamate-*meso*-diaminopimelate muropeptidase	**−2.3**	**−2.3**
Q88V80	*lp*_2197	*murD*	UDP-*N*-acetylmuramoylalanine--d-glutamate ligase	−1.1	**1.5**
F9UTQ8	*lp*_0304	*-*	Extracellular transglycosylase	1.0	**−2.6**
F9ULL9	*lp*_3676	*-*	Cell surface protein, CscC family	−1.9	**−2.1**
F9UME2	*lp*_0946	*-*	Mucus-binding protein, LPXTG-motif cell wall anchor	−3.7	**−3.0**
F9US24	*lp*_2940	*-*	Cell surface protein, LPXTG-motif cell wall anchor	−1.5	**−2.3**
WP_045352817.1*	*lp*_2401/*lp*_0681	*-*	Prophage P1/P2a protein/LysM peptidoglycan-binding domain-containing protein*	NA	**−3.1**

Comparing flow biofilm cells to static biofilm cells showed more differences in protein expression for both strains. For WCFS1, 116 proteins (7 %) were higher expressed and 110 proteins (6.6 %) lower expressed in flow biofilm cells compared to static biofilm cells (Fig. 5B). Higher expressed proteins were related to (myo)inositol metabolism, oxidoreductases/ubiquinone biosynthesis, metal binding, DNA repair, riboflavin production, cell wall formation/cell division and pyrimidine/purine metabolism (indicated with different colours in Fig. 5B, full overview of proteins can be found in supplementary data S3). Interestingly, two of the highest expressed proteins in flow were AsnB2 (∼141 fold) and AnsB (∼18 fold) (Table 1), which are both related to the conversion of l-aspartate to either l-asparagine or fumarate, respectively. Another protein of interest was the higher expressed cystathionine beta-synthase (Table 1). The lower expressed proteins include proteins related to sugar metabolism, purine metabolism, general stress, cell surface/adhesion and redox stress. In addition nine transcriptional regulators and ten transporter systems were differentially expressed in flow compared to static biofilm cells for WCFS1, indicating a change in regulation/uptake systems. An additional protein of interest is the histidine protein kinase (sensor protein) PlnB, which is part of a two-component system regulating bacteriocin production and was lower expressed in flow. Interestingly, both static biofilm and flow biofilm cells of WCFS1 showed a reduction of foldase proteins (PrsA and PrsA1, respectively) compared to supernatant cells, indicating a potential link for this protein secretion system to biofilm formation in general. Furthermore, 25 proteins related to the cell wall were found to be differentially expressed in WCFS1 flow biofilm cells, of which three overlapped with the surface proteins found in static biofilm cells, namely F9UP60, F9UUA0 and F9USE1 (−9.1, 1.7 and −2.3 fold respectively, Table 2).

For CIP104448, 79 proteins (5 %) were higher expressed and 85 proteins (5.4 %) were lower expressed in flow compared to static biofilm cells (Fig. 5D). Although more hypothetical proteins were found, the proteins that could be annotated match similar processes as observed in WCFS1 in flow biofilms cells compared to static. Proteins that were higher expressed were related to redox, riboflavin production, metal binding, cysteine/methionine metabolism, regulators, DNA repair, stress, biosynthesis of peptidoglycan and ribosomal proteins. Similarly, the two highest expressed proteins, a cysteine synthase family protein (WP_045351456.1) and SWIM zinc finger family protein (WP_045352415.1), were also found to be higher expressed in WCFS1 flow biofilm cells (Table 1). Lower expressed proteins include proteins related to oxidative stress such as catalases and thioredoxin, stress response, purine metabolism and proteins related to the cell wall/with peptidoglycan binding domains, including F9ULL9, which was already lower expressed in static biofilm cells as well (Table 2).

When comparing WCFS1 and CIP104448 flow versus static biofilm cells, both showed a metabolic change in the purine pathway from IMP towards GTP/ATP (WCFS1 expression of the purine pathway in flow can be found in Supplementary material S4). In addition both showed higher protein values of proteins related to riboflavin production and cysteine metabolism (Table 1), but lower expression of cell wall and membrane anchored proteins (Table 2). Interestingly, of the cell wall/surface and membrane anchored proteins, with the exception of F9USE1 and F9UUA0, different proteins were found for both strains in flow biofilm cells compared to static biofilm cells. This suggests that the addition of flow influences different cell surface proteins in both strains. Only F9UUA0, an extracellular protein, gamma-d-glutamate-*meso*-diaminopimelate muropeptidase, was lower expressed for both strains in both static and flow biofilm cells.

## 4 Discussion

The current study provides insight how flow affects the formation of biofilms in *Lactiplantibacillus plantarum* strains WCFS1 and CIP104448 using a comparative phenotyping and proteomics approach that revealed similar and unique responses for the two strains.

### 4.1 Static biofilms

The static biofilms of both strains are located at the bottom of the wells, likely due to sedimentation of the non-motile cells (Fig. 1A). There is a difference in CV-values between both strains, although no significant difference was observed between the cell numbers determined by plating (CFU/ml). Crystal violet binds not only to cells, but also to extracellular material, suggesting that CIP104448 static biofilm consists of more extracellular material compared to WCFS1. Research by Ramirez et al., 2015 showed that biofilm formation for WCFS1 from 24 h to 72 h increased the CV-values and corresponded with a decrease in cell counts, indicating cell lysis and extracellular material to be an important factor for WCFS1 biofilm development.

Indeed, in our 24 h biofilm, flow cytometry analysis of biofilm cells after removal of the extracellular DNA, shows that the static biofilm of WCFS1 consists of mostly (>98 %) live/intact cells, whereas the static biofilm of CIP104448 consists of ∼30 % dead and/or damaged cells with compromised membranes. Since CV stains live, dead and damaged cells, this could explain the higher CV values of CIP104448 compared to WCFS1. Although, CIP104448 shows more damaged/dead cells compared to WCFS1, the cell clusters were not influenced by DNase and Proteinase K treatments (Supplementary material S2), indicating reduced enzyme accessibility and/or that the biofilm matrix consists of other components besides eDNA and proteinous material. Finally, the SEM images highlight that both strains formed structurally very different biofilms. Whilst WCFS1 biofilm cells are aligned closer together, CIP104448 biofilm cells appear slightly elongated and mostly attached to each other at the poles which allows for a more open ‘3D’ structure of cell agglomeration of the biofilm. Altogether this shows that although the location of the static biofilm formation may be similar for both strains, the resulting biofilms formed by the two strains are different.

### 4.2 Flow biofilms

Flow can significantly influence biofilm formation and can alter biofilm development and cell physiology [48], which is also what we observed. Although both strains showed an increase in cell numbers of the flow biofilms compared to static biofilms, which is conceivably linked to the increased availability of nutrients with flow, there was a difference in their ability to form biofilm as indicated by the CV values (Fig. 1A–B). Applying flow to the wells allows for the normally non-motile cells to potentially colonize a larger surface area [49], as was observed for CIP104448 in flow where the biofilm was both on the bottom and sides of the well. This matches the higher hydrophobicity of CIP104448 compared toWCFS1 (Fig. 1F). Hydrophobic bacteria are more likely to attach to a hydrophobic surface than hydrophilic bacteria [50], which also explains the quicker attachment time of CIP104448 compared to WCFS1 to the hydrophobic polystyrene wells. Furthermore, the fluorescence images clearly show CIP104448 cells form agglomerates which could be related to higher hydrophobicity of the cells [51]. Interestingly, the composition of live, dead and damaged cells is mostly strain dependent and does not significantly differ between the supernatant and both types of biofilms (Fig. 2A). A difference in population composition of live, dead and damaged cells was expected between static and flow biofilms, as cell lysis has been reported to be an important factor in biofilm formation [23,28] and CV values changed with the addition of flow. Cell lysis may be increased by a lower pH [23], which can be neutralized by the addition of flow [52], and should be considered in future work. Furthermore, although the influence of hydrodynamics and nutrients on biofilm structure has been reported before [53,54], no clear influence of flow on the biofilm structure or population composition is observed for either strains in the SEM images, besides the increase in cell density.

### 4.3 Proteomes of static and flow biofilms

Based on the limited protein changes between the static biofilm cells and static supernatant cells for both strains and location of the biofilms, we conclude that formation of *L. plantarum* static biofilms does not involve activation of cellular processes previously described to be involved in bacterial biofilm formation including adaptive stress response and production of extracellular polysaccharides [28]. Our results match earlier findings by Ramírez et al., 2018, who showed for WCFS1 that an increase in CV-stained static biofilm formation correlated with a decrease in CFUs and enhanced cell lysis resulting in release of eDNA conceivably acting as matrix component [28]. Notably, in our experiments, changes in protein levels in static biofilm cells for both strains were related to the cell wall, including three proteins (F9ULM2, F9USE1 and F9UTQ7) that were between 3- and 63-fold lower expressed in both strains. Bacterial cell surfaces can play an important role in biofilm formation [51]. The cell surface protein F9ULM2 belongs to the CscB family, which has been proposed to play a role in carbon source acquisition [55] and is therefore more likely to be related to a different lifestyle than biofilm formation. However, the other two overlapping proteins are extracellular transglucosylases with a LysM peptidoglycan binding domain and are predicted to have an enzymatic function related to the biosynthesis or degradation of polysaccharides, a potential biofilm matrix component [56]. Future work should take into account biochemical analysis of extracellular polysaccharide production to elucidate possible roles in *L. plantarum* biofilm formation.

Biofilm formation is regulated via environmental signals and the addition of flow not only removes waste products and excreted molecules, but also replenishes nutrients including glucose, a known signal molecule that influences biofilm development in Gram-positive bacteria [57]. For the flow biofilms, most proteins can be attributed to changes in metabolic processes related to the higher availability of nutrients with flow. These changes were characterized by an increase in proteins related to cell wall/peptidoglycan formation, as well as cell division (as supported also by the higher CFU/ml). Additionally, there was a shift in purine/pyrimidine metabolism from inosine monophosphate (IMP) production towards Guanosine 5′-triphosphate/Adenosine 5′-triphosphate (GTP/ATP). Furthermore, (general) stress proteins were lower expressed, but redox/electron transfer such as oxidoreductases, metal binding proteins, and ubiquinone biosynthesis increased. Both strains also showed increased levels of proteins related to riboflavin production in flow compared to static biofilm cells, which can be related to the higher expression towards GTP with flow, which can continue towards riboflavin production (Supplementary material S4). Tolar et al.], found that riboflavin can support iron reduction, indicating electron transfer through a flavin-dependent route in *L. plantarum* [58], an important energy pathway when exposed to oxygen, which can be the case for flow biofilm cells due to the continuous supply of (aerated) fresh media in these conditions. In addition, applying flow caused various regulators and transport systems to be differentially expressed in both strains, suggesting a change in metabolic activity of the cells. Similarly, the substantial increased expression of AsnB2 (F9USK0, 140.1 fold) and AnsB (F9URT8, 17.6 fold) could be related to the increased glucose availability in flow conditions. Both proteins convert l-aspartate to l-asparagine or fumarate, respectively, which are both directly or indirectly part of the citrate cycle and can be related to the higher cell counts found in flow biofilm. Overall, the higher nutrient availability with flow imaginably can lead to a substantial portion of cells to be in exponential (fast growing) cell state compared to stationary (stable and slow growing), as is expected in static conditions with depleted nutrients. Similar to this, WCFS1 showed an upregulation of proteins related to myo-inositol metabolism with flow. Inositol has been related to increased resistance to stress in *L. plantarum* [59], but overall stress proteins go down for WCFS1 with flow in our data, suggesting less stress for the cells with flow. The myo-inositol cluster which includes the six higher expressed proteins (lp_3604–3615), was previously found to correspond to growth of *L. plantarum* on different sugars and has also been hypothesised to be involved in the metabolism of various different sugar alcohols [60]. Therefore, we hypothesize that the higher expression of myo-inositol production in the tested conditions is related to the increased glucose availability with flow.

### 4.4 Biofilm resistance to the disinfectant peracetic acid

Besides the resistance against DNase and Proteinase K, we also tested the resistance against PAA on planktonic cells, cells in biofilms and resuspended cells from biofilms formed under static and flow conditions (Fig. 4). PAA is a commonly used disinfectant conceivably acting as an oxidizing agent targeting various cell components such as the cell membrane and enzymes [61]. Biofilm cells are often more resistant to disinfectant treatments, such as PAA, than planktonic cells [25,62], which was also the case for our results. Greater resistance of cells in a biofilm compared to planktonic cells could be due to limited access and/or binding of the disinfectants to the matrix components reducing their effectiveness in the latter condition. In addition, based on the SEM images and DNase/protease effects, the ‘dense packaging’ of the biofilm cells may offer another explanation of the reduced effectivity of PAA against biofilm cells. It should be noted that the cell suspensions were not standardized prior to the disinfectant exposure and thus corresponded to the cell numbers indicated in Fig. 1C. Higher cell counts, as found in the biofilms, can reduce the effectiveness of disinfectants, however data comparison and correlation analysis (Supplementary material S5) indicated that this is not the case in our tested conditions. Combining all data, no correlation was found and the dispersed static biofilm had the same cell concentration as the intact static biofilm, but the dispersed static biofilm was more susceptible to PAA. For both strains, the increased disinfectant sensitivity of the dispersed static biofilms is likely due to the disruption of the protective biofilm layer and organic matter that can protect cells against disinfectants [63]. Although the exact mode of action of PAA is not yet fully understood, it is hypothesised to work on the cell membrane/wall and research by Zhang et al. [62], has shown that PAA created holes in the centre of the cells. Although the proteome data revealed changes in the expression levels of proteins related to the cell wall of both strains, it remains unclear if and how they may have contributed to the increased resistance of the biofilm cells compared to planktonic cells. For CIP104448, the resistance to PAA is overall higher in all fractions tested compared to WCFS1, highlighting the strain variability.

Interestingly, for WCFS1, the biofilm grown with flow was more resistant compared to the static biofilm cells, and for CIP104448 maintained a similar high resistance. For CIP104448, even dispersion of the flow biofilm did not diminish this resistance. The addition of flow likely results in more cells in the logarithmic phase as nutrients, especially glucose, are constantly supplied with the fresh media which can be observed from the before mentioned proteome differences in metabolic processes as well. In addition, WCFS1 dispersed flow biofilm showed similar sensitivity to PAA as the supernatant cells, suggesting that the biofilm matrix and not the state of the cells to be the protective factor for WCFS1. Interestingly, CIP104448 flow biofilms do not display an increased sensitivity to the disinfectant when disrupted and small aggregates are released. Potentially, the CIP104448 cell aggregates could allow for less contact with the disinfectant and subsequently lead to higher resistance. However, this is not the case for dispersed static biofilm cells which also form aggregates but do show sensitivity to PAA. Therefore, not only the structure of the biofilm or aggregation of CIP104448 cells but, also the metabolic cell state plays an important role in increased PAA resistance. We hypothesize that the limited sensitivity to the disinfectant of dispersed CIP104448 flow biofilm cells can be attributed to an increase in stress proteins including proteins related to riboflavin production, metal-binding, oxidative stress defense and DNA repair that were higher expressed in these cells. Whether (dispersed) *L. plantarum* WCFS1 and CIP104448 flow biofilms show resistance to other types of disinfectants remains to be elucidated.

In conclusion, combining all results highlights that *L. plantarum* biofilm structure and matrix, physiological cell state and stress resistance of the cells is strain dependent and strongly affected in flow conditions. Further studies using dynamic flow conditions, along with strains isolated from food processing environments and foods, are recommended to aid in better understanding *L. plantarum* biofilm formation in food industry settings.

## CRediT authorship contribution statement

**Linda Huijboom:** Writing – review & editing, Writing – original draft, Visualization, Validation, Project administration, Methodology, Investigation, Formal analysis, Data curation, Conceptualization. **Parisa Rashtchi:** Writing – review & editing, Writing – original draft, Visualization, Validation, Project administration, Methodology, Investigation, Formal analysis, Data curation, Conceptualization. **Marcel Tempelaars:** Writing – review & editing, Validation, Supervision, Project administration, Methodology, Investigation, Formal analysis, Data curation, Conceptualization. **Sjef Boeren:** Writing – review & editing, Software, Formal analysis, Data curation. **Erik van der Linden:** Writing – review & editing, Supervision, Resources, Project administration, Funding acquisition. **Mehdi Habibi:** Writing – review & editing, Validation, Supervision, Resources, Project administration, Funding acquisition. **Tjakko Abee:** Writing – review & editing, Validation, Supervision, Resources, Project administration, Methodology, Investigation, Funding acquisition, Conceptualization.

## Declaration of competing interest

The authors declare that they have no known competing financial interests or personal relationships that could have appeared to influence the work reported in this paper.

## Data Availability

Data will be made available on request.
